# Sugar Allocation to Metabolic Pathways is Tightly Regulated and Affects the Virulence of *Streptococcus mutans*

**DOI:** 10.3390/genes8010011

**Published:** 2016-12-28

**Authors:** Miki Kawada-Matsuo, Yuichi Oogai, Hitoshi Komatsuzawa

**Affiliations:** Department of Oral Microbiology, Kagoshima University Graduate School of Medical and Dental Sciences, Kagoshima 890-8544, Japan; mmatsuo@dent.kagoshima-u.ac.jp (M.K.-M.); oogai@dent.kagoshima-u.ac.jp (Y.O.)

**Keywords:** *Streptococcus mutans*, GlmS, NagB, sugar distribution, virulence

## Abstract

Bacteria take up and metabolize sugar as a carbohydrate source for survival. Most bacteria can utilize many sugars, including glucose, sucrose, and galactose, as well as amino sugars, such as glucosamine and *N*-acetylglucosamine. After entering the cytoplasm, the sugars are mainly allocated to the glycolysis pathway (energy production) and to various bacterial component biosynthesis pathways, including the cell wall, nucleic acids and amino acids. Sugars are also utilized to produce several virulence factors, such as capsule and lipoteichoic acid. Glutamine-fructose-6-phosphate aminotransferase (GlmS) and glucosamine-6-phosphate deaminase (NagB) have crucial roles in sugar distribution to the glycolysis pathway and to cell wall biosynthesis. In *Streptococcus mutans*, a cariogenic pathogen, the expression levels of *glmS* and *nagB* are coordinately regulated in response to the presence or absence of amino sugars. In addition, the disruption of this regulation affects the virulence of *S. mutans*. The expression of *nagB* and *glmS* is regulated by NagR in *S. mutans*, but the precise mechanism underlying *glmS* regulation is not clear. In *Staphylococcus aureus* and *Bacillus subtilis*, the mRNA of *glmS* has ribozyme activity and undergoes self-degradation at the mRNA level. However, there is no ribozyme activity region on *glmS* mRNA in *S. mutans*. In this review article, we summarize the sugar distribution, particularly the coordinated regulation of GlmS and NagB expression, and its relationship with the virulence of *S. mutans*.

## 1. Introduction

*Streptococcus mutans* is a commensal bacterium in the human oral cavity and is a well-known cariogenic pathogen [1]. *S. mutans* has a key role in the formation of biofilms (dental plaque), which underlie several major oral diseases and tooth decay. This organism produces glucosyltransferases (GTFs), which are involved in the production of a water-insoluble, sticky glucan through the use of sucrose as the sole substrate [1,2]. This insoluble glucan is responsible for biofilm formation. In addition, acid production is a virulence factor in *S. mutans*, as acid demineralizes the tooth surface leading to tooth decay [2,3]. Acids are metabolites in the Embden–Meyerhof carbon metabolic pathway, which is important in ATP generation. *S. mutans* also synthesizes intercellular polysaccharides (IPSs) when there is an excess of carbon and utilizes these IPSs in ATP production when carbon sources are limited in the environment. Therefore, it is clear that carbohydrates are very important in the virulence of *S. mutans*.

Sugars are the major carbohydrate sources used by bacteria. Bacteria can incorporate several sugars in the cytoplasm and utilize them for ATP production through glycolysis, and synthesis of bacterial components (peptidoglycan, lipoteichoic acid and nucleic acids) and polysaccharides [4,5,6,7]. There are many types of sugars, including monosaccharides (glucose, fructose, mannose), disaccharides (sucrose, lactose, maltose), trisaccharides (raffinose), and amino sugars (glucosamine, *N*-acetylglucosamine) in the environment/host. The types of sugars utilized depend on the bacterial species [8,9]. *S. mutans* is known to utilize raffinose as a carbon source, whereas other streptococci cannot utilize it [10,11]. When multiple sugars are present in the environment, bacteria can utilize several sugars. We have previously demonstrated that sugars and amino sugars are utilized and distributed into pathways for glycolysis and bacterial component biosynthesis pathways in *S. mutans* [12]. The disturbance of this regulation results in alterations in the virulence of the bacteria. Furthermore, catabolite control protein A (CcpA), which regulates the expression of many factors involved in sugar metabolism, also affects the virulence of *S. mutans* and *Staphylococcus aureus* [13,14,15]. Therefore, sugar metabolism is very important not only in bacterial physiology but also in the virulence of bacteria.

In this review article, we summarize the incorporation, distribution, and metabolism of sugars in *S. mutans*. In particular, we focus on the factors affecting the allocation of sugars and amino sugars to the glycolysis and cell wall biosynthesis pathways and on the association of sugar metabolism with virulence in *S. mutans*.

## 2. *S. mutans* Virulence Is Related to Sugar Metabolism

**Figure 1:**
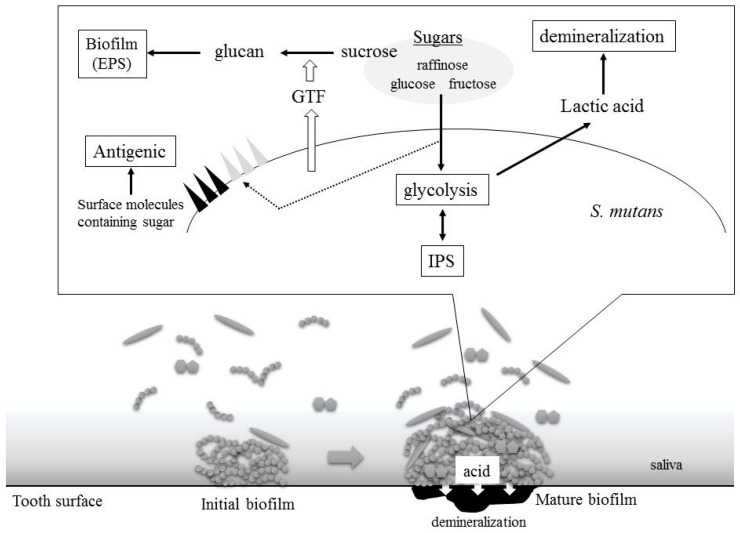
*Streptococcus mutans* virulence is related to sugars. GTF: glucosyltransferase; IPS: intercellular polysaccharide; EPS: extracellular polysaccharide.

In *S. mutans*, sugars are utilized both intracellularly and extracellularly. Several virulence factors are generated through sugar metabolism in *S. mutans* (Figure 1). Internal sugars are primarily used for glycolysis, biosynthesis of various components, such as cell walls and lipoteichoic acid (LTA), and IPS biosynthesis. In addition, external sucrose is utilized for the production of extracellular polysaccharides (EPSs) known as glucans.

### 2.1. Acid Production

**Figure 2:**
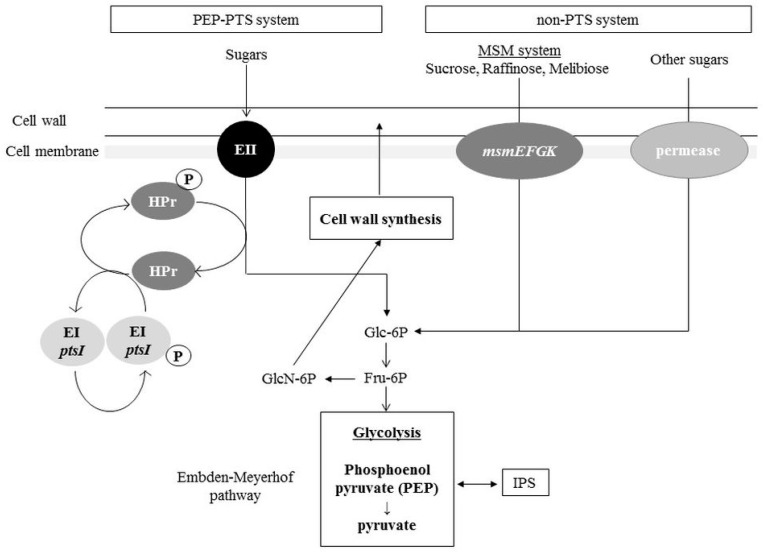
Phosphotransferase (PTS) and non-PTS system in *S. mutans*. Bacteria can take up carbohydrates by specific sugar incorporation systems. In *S. mutans*, two major incorporation systems, the PTS and non-PTS systems, have been reported. After sugars are incorporated into cytoplasm, the sugar molecule is processed and finally metabolized to fructose 6-phosphate (Fru-6P). Fru-6P is distributed to glycolysis and the cell wall synthesis system. EI: Enzyme I; EII: Enzyme II; MSM: multiple sugar metabolism; msmEFGK: multiple sugar metabolism transporter; Glc-6P: glucose 6-phosphate; GlcN-6P: glucosamine 6-phosphate; HPr: histidine phosphocarrier protein.

Acid, primarily lactic acid, is a major virulence factor in *S. mutans* and causes demineralization of calcium phosphate in teeth [2,3]. Acids are produced by the glycolysis pathway, which is a biochemical reaction that occurs in the cytoplasm. Since glycolysis is the most primitive metabolic system for energy acquisition, almost all organisms have this system. Glucose is the most accessible carbon source for many bacteria. Through this metabolic process, glucose is decomposed into organic acids such as pyruvic acid, lactic acid, and formic acid. ATP is also produced by this process. In *S. mutans*, incorporated sugars are processed via the Embden-Meyerhof pathway and metabolic degradation (Figure 2). In the Embden-Meyerhof pathway, phosphorylated glucose is metabolized to fructose 6-phosphate, from which acids are ultimately produced. In dental plaque, *S. mutans* produces, primarily, lactic acid and causes acid accumulation. When the pH is below 5.5, demineralization of the tooth surface occurs and causes tooth decay.

### 2.2. Biofilm Formation

The strong association of dental plaques with tooth decay and periodontal diseases is well accepted. Dental plaque is also called a biofilm because dental plaque consists of many types of bacteria and their extracellular matrix (ECM) products. The major ECM component in dental plaque is a sticky, water-insoluble glucan produced by *S. mutans*. *S. mutans* produces three GTFs and synthesizes a sticky, water-insoluble extracellular glucan from sucrose as the sole substrate [16,17]. This glucan is a complex of water-insoluble (glucose polymer with an α-1,3 linkage) and soluble glucan molecules (glucose polymer with an α-1,6 linkage) termed mutan and dextran, respectively. This sticky glucan causes strong adhesion and bacterial aggregation on the tooth surface, facilitating biofilm formation. The glucan also serves as a stored carbon source in *S. mutans*. *S. mutans* produces dextranase to digest glucan (dextran) to glucose, which can then be utilized in glycolysis [18,19,20]. In addition, *S. mutans* produces fructosyltransferases for the production of a fructose polymer known as fructan, which is a water-soluble polysaccharide.

### 2.3. Intercellular Polysaccharides Synthesis

To maintain its carbon sources, *S. mutans* synthesizes IPSs, together with EPSs, by using sugars present in the environment. IPSs in *S. mutans* are polysaccharides of glucose (glucan) with α1-4 glycosidic bonds [21]. The glucose 1-phosphate (Glu-1P) generated from glucose 6-phosphate is activated with ATP and becomes ADP-glucose, which is incorporated into the IPS primer. When the environmental carbohydrates are limiting, IPSs are processed to glucose 1-phosphate after phosphorolysis and are metabolized back through the glycolysis system.

### 2.4. Cell Surface Antigens: Serotype-Specific Carbohydrate Antigens and Lipoteichoic Acids

In *S. mutans*, carbohydrate antigens known as serotype-specific antigens are localized in the cell wall. *S. mutans* produces rhamnose–glucose polysaccharides (RGPs) composed of α1,2- and α1,3-linked rhamnan backbones and glucose side-chains that are linked to alternate rhamnose molecules in the cell wall [22]. The differences in the composition of the RGP sugar chain are used as the serotype carbohydrate antigens for classifying *S. mutans* [23]. Among *S. mutans* strains, four serotypes (c, e, f and k) have been reported [24]. Serotype-specific antigens have been reported to be involved in platelet aggregation [25].

Lipoteichoic acids (LTAs) are antigenic molecules in Gram-positive bacteria. LTA is an amphiphilic molecule consisting of hydrophilic polysaccharides and hydrophobic glycolipids. The structure of *S. mutans* LTA consists of a diacylated glycolipid anchor linked to poly-glycerophosphate units esterified with d-alanine or d-*N*-acetylglucosamine. *S. mutans* LTA has been demonstrated to induce the expression of inflammatory mediators in murine macrophages [26] and to induce apoptosis in cultured human dental pulp cells [27].

## 3. Distribution Pathways for Sugars and Amino Sugars into the Glycolysis Pathway and Cell Wall Synthesis Pathways in *S. mutans*

Carbohydrates are important for both normal cell physiology and virulence in *S. mutans*. Therefore, *S. mutans* can internalize many types of sugars into the cytoplasm via several uptake systems, thus allowing it to use various metabolic pathways. In addition, the incorporated sugars are coordinately used for cell wall biosynthesis, glycolysis, and the synthesis of several molecules containing sugars.

### 3.1. Sugar Incorporation: PTS and Non-PTS Systems

Bacteria can take up carbohydrates, including sugars and amino sugars. Since sugars are relatively large molecules, they cannot pass through the cell membrane via diffusion. Thus, specific sugar incorporation systems are present in bacteria [28]. In *S. mutans*, two major incorporation systems have been reported:

(1) PTS system: Phosphoenol pyruvate-dependent phosphotransferase. The phosphoenol pyruvate-dependent phosphotransferase (PEP-PTS) system is involved in the uptake of sugars and sugar derivatives such as sugar alcohols and amino sugars. The PEP-PTS system consists of three components: enzyme I (EI), EII (EIIA-C) and histidine-containing phosphocarrier protein (HPr). EI and HPr are generally present as cytoplasmic components, which are related to all PTSs for carbohydrate uptake. However, the EII proteins are specific for one sugar or a group of closely related sugars [28]. In the case of glucose uptake, glucose binds to EII and is incorporated into the cell membrane where it is phosphorylated by phosphorylated HPr (P-HPr), thus forming glucose 6-phosphate, which then immediately enters the glycolysis pathway. Through this process, PEP phosphorylates EI and becomes pyruvic acid after losing a phosphate group (Figure 2). Then, phosphorylated EI phosphorylates HPr, which adds phosphate to the sugar.

In *S. mutans*, a genomic analysis has revealed 14 sets of PTSs with a variety of EII domains, suggesting that *S. mutans* can metabolize various types of sugars [29]. Several PTSs in *S. mutans* have already been investigated in detail [30,31,32,33,34]. Ajdić et al. have reported a comprehensive analysis of PTSs using various sugars in *S. mutans* and have observed that six PTSs were transcribed in response to specific sugars present in the medium, whereas the other PTSs were constitutively expressed or repressed under all conditions tested [29]. Moye et al. have demonstrated that PTSs consisting of ManLMN and glucose/mannose enzyme II permease are associated with the uptake of amino sugars such as glucosamine (GlcN) and *N*-acetylglucosamine (GlcNAc) [30].

(2) Non-PTS system: Binding protein-dependent transport. Whereas PEP-PTSs have high sugar specificity and affinities, binding protein-dependent transport systems (BPTS) have low sugar specificity and can transport multiple types of sugars, including high molecular weight sugars such as trisaccharides. In *S. mutans*, the multiple sugar metabolism (MSM) system has been well characterized [35]. The MSM system consists of a transport protein complex containing a membrane protein (MsmFG), a sugar-binding protein (MsmE) and an ATP-binding protein (MsmK). MsmEFGK is able to transport melibiose, raffinose, isomaltose, and isomaltoriose. BPTS transports the sugar into the cytoplasm by using the energy from ATP hydrolysis (Figure 2). The transported disaccharide or trisaccharide is metabolized by hydrolytic enzymes in the bacterial cytoplasm.

### 3.2. Allocation of Sugars and Amino Sugars to Glycolysis and Cell Wall Biosynthesis

**Figure 3:**
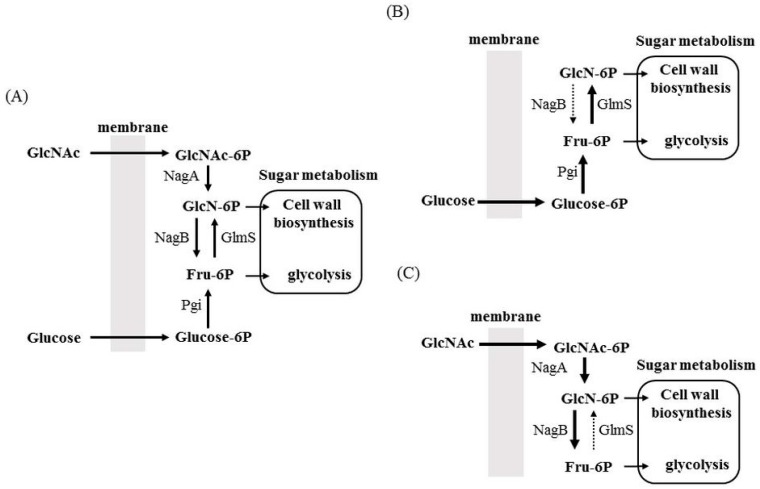
Proposed sugar distribution mediated by glutamine-fructose-6-phosphate aminotransferase (GlmS) and glucosamine-6-phosphate deaminase (NagB). (**A**) In the presence of sugar (glucose) and amino sugar (GlcNAc), both sugars are incorporated into the cytoplasm and are distributed to cell wall synthesis and glycolysis by GlmS and NagB; (**B**) In the presence of glucose as a sole carbohydrate source, NagB expression is significantly suppressed to generate enough GlcN-6P for the cell wall biosynthesis pathway; (**C**) In the presence of GlcNAc as a sole carbohydrate source, GlmS expression is significantly suppressed to generate enough Fru-6P for glycolysis pathway. GlcNAc: *N*-acetylglucosamine; GlcNAc-6P: *N*-acetylglucosamine 6-phosphate; GlcN-6P: glucosamine-6-phosphate; Fru-6P: fructose 6-phosphate; glucose-6P: glucose 6-phosphate; NagA: *N*-acetylglucosamine 6-phosphate deacetylase; Pgi: glucose 6-phosphate isomerase.

When sugars are incorporated into the cytoplasm through the PTS system, the sugars are phosphorylated and are primarily metabolized into fructose 6-phosphate (Fru-6P) for glycolysis and cell wall peptidoglycan (PG) biosynthesis (Figure 3). To process Fru-6P for glycolysis, Fru-6P is converted to fructose 1,6-biphosphate. Fru-6P is also converted to glucosamine 6-phosphate (GlcN-6P) for PG biosynthesis. Amino sugars such as glucosamine and *N*-acetylglucosamine are incorporated into the cytoplasm in their phosphorylated form by the PTS system and are then processed to GlcN-6P. The generated GlcN-6P is utilized for PG biosynthesis and may also be converted to Fru-6P for glycolysis (Figure 3A). Therefore, the distribution of Fru-6P and GlcN-6P plays a crucial role in bacterial physiology. Glutamine-fructose-6-phosphate aminotransferase (GlmS) and glucosamine-6-phosphate deaminase (NagB) play a central role in the distribution of sugars and amino sugars into the glycolysis or cell wall biosynthesis pathways [5,36]. GlmS catalyses the production of GlcN-6P from Fru-6P, and NagB is important in the production of Fru-6P from GlcN-6P. Therefore, these enzymes possess opposing activities. We have previously demonstrated that the expression of *glmS* and *nagB* is coordinately regulated [12]. When amino sugars (GlcNAc) are not present in medium containing glucose, *glmS* is highly expressed, whereas *nagB* is not expressed (Figure 3B). In contrast, when amino sugars are the only sugar source in the medium, *glmS* expression is significantly repressed, and *nagB* expression is significantly increased (Figure 3C). Thus, sugar and the amino sugar incorporation can be readily switched through the expression of *glmS* and *nagB*. Interestingly, these expression patterns are regulated by the ratio of GlcNAc to glucose. When the GlcNAc concentration is gradually increased in the medium containing glucose, *glmS* expression is gradually suppressed, whereas *nagB* expression is gradually increased. Therefore, *glmS* and *nagB* are regulated by the ratio of glucose to GlcNAc, thus allowing optimal glycolysis and cell wall synthesis to be maintained (Figure 3).

We also have constructed and characterized *glmS* and *nagB* knockout mutants [12]. We have observed significant growth inhibition in the *glmS* mutant grown in the absence of sufficient GlcNAc and in the *nagB* mutant grown in the presence of GlcNAc. In the *glmS* mutant, the supply of GlcN-6P converted from Fru-6P is abolished in the absence of GlcNAc, thus resulting in complete inhibition of cell wall biosynthesis and leading to cell death. In the *nagB* mutant grown in the presence of high GlcNAc concentrations, the amount of Fru-6P is significantly decreased due to insufficient processing and the accumulation of toxic GlcN-6P leads to cell death. These results indicate that both enzymes are essential under specific conditions (with or without GlcNAc) in *S. mutans*.

## 4. Factors Regulating *nagB* and *glmS* Expression

### 4.1. NagR

**Figure 4:**
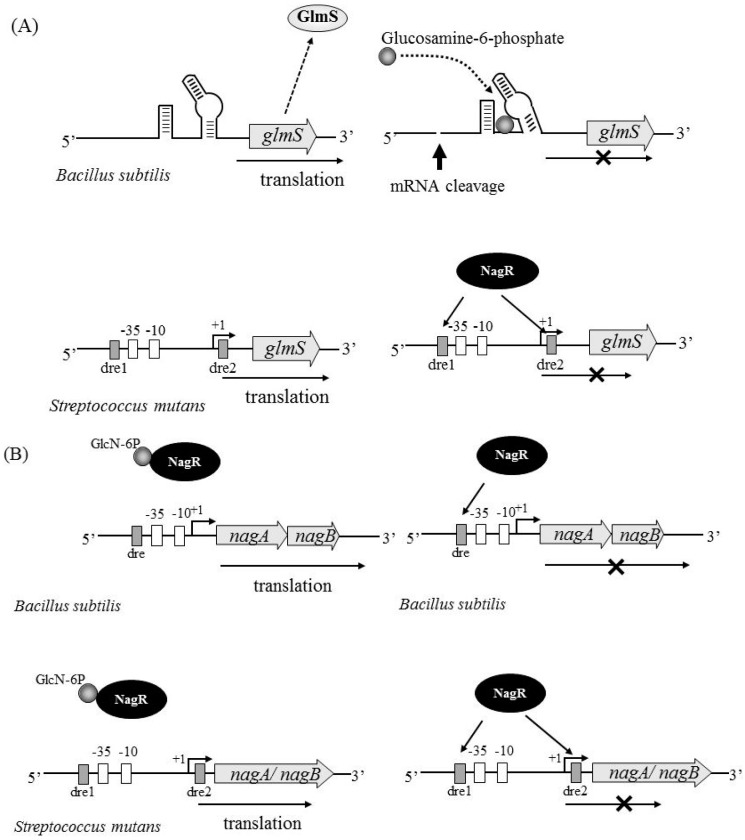
Regulation of *glmS* and *nagAB* expression in *Bacillus subtilis* and *S. mutans*. (**A**) The regulation of *glmS* in *B. subtilis* (upper) and *S. mutans* (lower) is shown. *B. subtilis* *glmS* transcript has ribozyme activity, which causes self-cleavage in the presence of excess glucosamine-6-phosphate, a product of the GlmS reaction. GlmS of *S. mutans* is negatively regulated by NagR. NagR binds to the DasR responsive element (dre) sequence upstream of the *glmS* coding region, leading to inhibition of the *glmS* transcript; (**B**) Regulation of *nagAB* in *B. subtilis* (upper) and *S. mutans* (lower) is shown. In *B. subtilis*, *nagA* and *nagB* are tandemly located to form an operon, while *nagA* and *nagB* are independently located in *S. mutans*. NagR binds to the dre sequence upstream of *nagAB* in *B. subtilis*, leading to inhibition of the *nagAB* expression (upper right). However, NagR bound with GlcN-6P has no ability to bind to the dre sequence (upper left). The same regulation system is observed in *nagA* and *nagB* in *S. mutans* (lower left and right).

GlmS regulation has been well characterized in several bacterial species. Of note, the *glmS* transcript in *Bacillus subtilis* has ribozyme activity, which causes self-cleavage in the presence of excess GlcN-6P, a product of the GlmS reaction [37,38,39,40]. A consensus ribozyme core sequence is located 200–300 bp upstream of the *glmS* coding region in several Gram-positive bacteria, including *Bacillus* species and *S. aureus* (Figure 4A upper). This region of the mRNA can bind to GlcN-6P and mediate self-cleavage, therefore inhibiting *glmS* translation. In *S. mutans*, we have not found a consensus ribozyme sequence (homologous to the *B. subtilis* core region of the ribozyme) upstream of the *glmS* coding region. We have also investigated the transcriptional start site in *glmS* by rapid amplification of complementary DNA ends (RACE) experiments and have determined that the start site is 87 bp upstream of the *glmS* coding region [12]. These results suggest that *S. mutans glmS* does not have ribozyme activity.

Recently, NagR has been identified as a transcriptional regulator of *nagA*, *nagB*, and *glmS* expression [41,42]. NagR has homology to the GntR/HutC type regulator in some bacteria. In *B. subtilis*, *nagAB* expression was shown to be regulated by NagR (Figure 4B upper), while the regulation of *glmS* expression was shown to be independent of NagR (Figure 4A upper) [41]. Zeng et al. have found a DasR responsive element (dre) sequence upstream of *nagA*, *nagB*, and *glmS*, which has been reported to be the binding site of NagR in *S. mutans* [42]. They have clearly demonstrated NagR binding to the upstream sequence of each gene by electrophoretic mobility shift assay (EMSA), thus indicating that NagR directly regulates the expression of these genes (Figure 4A lower and 4B lower). They have also demonstrated that the tight regulation of *nagB* and *glmS* expression is perturbed in a *nagR* knockout mutant, which shows constitutively high expression of all three genes in the presence or absence of GlcNAc [42]. The mechanism by which only one transcriptional regulator, NagR, simultaneously regulates the expression of both *nagB* and *glmS* is unclear. There may be other factor(s) involved in regulating *nagB* and *glmS* expression.

### 4.2. Carbon Catabolite Repression

The carbohydrate metabolism of bacteria is usually under the control of carbon catabolite repression (CCR) [43] in which rapidly metabolizable carbohydrates, primarily glucose, repress the expression of factors involved in the metabolism of other carbohydrates. Catabolite control protein A (CcpA) [44,45] and the histidine phosphocarrier protein HPr [46] are generally believed to function in the regulation of CCR in most low-G+C Gram-positive bacteria. HPr phosphorylation (HPr-Ser-P) is triggered by fructose 1,6-bisphosphate [45], a glycolysis intermediate. In the presence of a preferred carbohydrate, i.e., glucose, HPr-Ser-P serves as a cofactor for facilitating CcpA binding to the promoter regions of the CCR-regulated genes.

Since expression of NagB and GlmS is affected by the ratio of glucose and GlcNAc, we have investigated the relationships between CcpA and the expression levels of these proteins [12]. We have analysed the expression of *glmS* and *nagB* in the *ccpA* mutant in the presence or absence of GlcNAc and have found no phenotypic alterations compared with wild type bacteria. We have found that *glmS* expression is decreased after the addition of GlcNAc, whereas *nagB* expression is increased. These results suggest that CcpA is not involved in regulating the expression of *glmS* or *nagB* under the tested growth conditions. In *S. mutans*, *manL*, which encodes the mannose/glucose permease EIIAB_L_, was demonstrated to be involved in CCR and the regulation of several virulence genes [47]. Generally, the HPr-CcpA complex directly participates in the classical CCR model for low G+C Gram-positive bacteria by binding to the promoter regions of genes that are under CCR [48]. However, this mechanism is not applicable for *S. mutans* because the lack of CcpA did not cause the alleviation of CCR-related genes [47,49]. Similarly, ManLMN was demonstrated to be a central metabolic regulator in *Streptococcus pneumoniae* [50]. Based on these results, NagB and GlmS may be related to the mannose/glucose PTS (man*LMN*).

## 5. Association of Carbohydrates with Virulence

We have investigated the association of NagB and GlmS with virulence in *S. mutans* [12]. GTFs, Pac (cell surface protein antigen), Gbp (glucan binding protein), and acid production are considered to be the major virulence factors in *S. mutans* (Table 1). Three GTFs have been shown to be involved in sucrose-dependent biofilm formation [51]. Among the GTFs, GTF-B, and GTF-C synthesize water-insoluble glucan, whereas GTF-D synthesizes water-soluble glucan. PAc is also a major virulence factor in *S. mutans* and is associated with the binding to the pellicle on the tooth surface [52]. We have found that the expression of GTFs (GTF-B and -C) and PAc is decreased in *nagB* mutants, whereas the expression levels are increased in *glmS* mutants. These results indicate that inactivation of *glmS* or *nagB* alters the expression of these virulence factors [12]. Biofilm formation has also been investigated in these mutants. The *nagB* mutant has decreased biofilm formation, whereas the *glmS* mutant has increased biofilm formation, indicating that the coordinate regulation of *glmS* and *nagB* expression is related to biofilm formation (Table 1). In addition to GTFs and PAc, GbpB, and AtlA (autolytic enzyme) have also been reported to be involved in biofilm formation [53,54]. Since GlmS and NagB are involved in biofilm formation, we have also investigated biofilm-related gene expression in the *glmS* and *nagB* mutants. The expression of *gbpB* and *gtfBC* has been found to be increased in the *glmS* mutant and decreased in the *nagB* mutant. However, *atlA* expression is not changed in either of the mutants [12] (Table 1). Furthermore, we have found increased salivary aggregation in the *glmS* mutant and decreased salivary aggregation the *nagB* mutant because PAc is responsible for sucrose-independent adherence to tooth surfaces and salivary aggregation, due to its binding to the salivary agglutinin gp340 [55].

Since GlmS or NagB are not considered to directly regulate virulence factors such as *gtfs*, *spaP* (PAc), and *gbpB*, the disruption of the coordinated regulation of NagB and GlmS might lead to the altered expression of other factors involved in the expression of virulence factor(s). It has been reported that one orphan response regulator (*gcrR*) and one two-component system (TCS) termed *vicR* are associated with *gtfB* and *gtfC* expression in *S. mutans* [56,57]. In addition, it has previously been reported that VicR, the response regulator of the TCS, regulates the expression of *gbpB* and is associated with the initiation of biofilm formation [58]. We have found that *vicR* expression is altered in both the *glmS* and *nagB* mutants, whereas *gcrR* expression is unchanged. From these results, we conclude that the altered virulence in the *glmS* and *nagB* mutants is caused by the alterations in VicR. These results suggest that failures in coordinate regulation of *glmS* and *nagB* affect bacterial virulence.

## 6. Conclusions

Carbohydrates are a major nutrient source for bacteria. Glucose is a good carbon source that is utilized for various metabolic pathways in the majority of microorganisms. However, because glucose can be scarce in the environment, bacteria can also metabolize various other types of sugars via the PTS and non-PTS systems. Therefore, the allocation of the sugars incorporated in various metabolic pathways is very important. In particular, amino sugars, including GlcN and GlcNAc, are abundant carbohydrates in the environment. To efficiently utilize amino sugars for energy production and biosynthesis of various bacterial components, many bacteria possess an amino sugar metabolic system. *S. mutans*, a commensal bacterium of the oral cavity, possesses a distribution system involving GlmS and NagB to efficiently produce ATP as an energy currency and to synthesize cell walls. This survival strategy is thought to have developed to allow *S. mutans* to adapt to environmental changes in the oral cavity that occur during the consumption of food and drink. In addition, sugar/amino sugar allocation is related to the virulence of *S. mutans*. It would be helpful to elucidate the detailed mechanisms by which the various sugars are metabolized by *S. mutans* to determine how bacteria colonize the human oral cavity. Furthermore, the research on sugar allocation in *S. mutans* may be applicable to other bacterial species, including other streptococci.

## Figures and Tables

**Table 1:** Effect of GlmS and NagB on virulence gene expression and phenotype.

	Gene Expression						Phenotype			
Inactivation Gene	*gtfB*	*gtfC*	*spaP*	*gbp*	*atlA*	*vicR*	Biofilm Formation	Salivary Aggregation	Growth	
									Glucose	GlcNAc
*glmS*	Up ^a^	Up	Up	Up	NC ^c^	Up	Up	Up	No	Yes
*nagB*	Down ^b^	Down	Down	Down	NC	Down	Down	Down	Yes	No

^a^ Increased expression or activity compared to the wild type. ^b^ Decreased expression or activity compared to the wild type. ^c^ Similar expression compared to the wild type.
